# More than two decades of research on insect neuropeptide GPCRs: an overview

**DOI:** 10.3389/fendo.2012.00151

**Published:** 2012-11-30

**Authors:** Jelle Caers, Heleen Verlinden, Sven Zels, Hans Peter Vandersmissen, Kristel Vuerinckx, Liliane Schoofs

**Affiliations:** Animal Physiology and Neurobiology, Department of Biology, Zoological InstituteKU Leuven, Leuven, Belgium

**Keywords:** insects, neuropeptides, G protein-coupled receptors, signal transduction, neurobiology

## Abstract

This review focuses on the state of the art on neuropeptide receptors in insects. Most of these receptors are G protein-coupled receptors (GPCRs) and are involved in the regulation of virtually all physiological processes during an insect's life. More than 20 years ago a milestone in invertebrate endocrinology was achieved with the characterization of the first insect neuropeptide receptor, i.e., the *Drosophila* tachykinin-like receptor. However, it took until the release of the *Drosophila* genome in 2000 that research on neuropeptide receptors boosted. In the last decade a plethora of genomic information of other insect species also became available, leading to a better insight in the functions and evolution of the neuropeptide signaling systems and their intracellular pathways. It became clear that some of these systems are conserved among all insect species, indicating that they fulfill crucial roles in their physiological processes. Meanwhile, other signaling systems seem to be lost in several insect orders or species, suggesting that their actions were superfluous in those insects, or that other neuropeptides have taken over their functions. It is striking that the deorphanization of neuropeptide GPCRs gets much attention, but the subsequent unraveling of the intracellular pathways they elicit, or their physiological functions are often hardly examined. Especially in insects besides *Drosophila* this information is scarce if not absent. And although great progress made in characterizing neuropeptide signaling systems, even in *Drosophila* several predicted neuropeptide receptors remain orphan, awaiting for their endogenous ligand to be determined. The present review gives a précis of the insect neuropeptide receptor research of the last two decades. But it has to be emphasized that the work done so far is only the tip of the iceberg and our comprehensive understanding of these important signaling systems will still increase substantially in the coming years.

## Introduction

The class of Insecta, which consists of more than 30 orders, forms the most diverse animal group on earth. With about one million documented species and presumably 10–30 million awaiting to be described, insects probably account for 50–70% of all existing animals (Scherkenbeck and Zdobinsky, 2009; Bellés, 2010; Van Hiel et al., 2010). Basically all the physiological processes during an insect's life cycle are regulated by neuropeptides, including developmental processes, behavioral functions, metabolic events and reproduction. As such, neuropeptides are the largest (very versatile) class of extracellular signaling molecules that are involved in communication between insect cells (Gäde and Goldsworthy, 2003; Meeusen et al., 2003; Claeys et al., 2005a). The insect neuropeptides and their actions have extensively been reviewed in the past (Nässel, 2002; Gäde and Auerswald, 2003; Gäde and Goldsworthy, 2003; Meeusen et al., 2003; Altstein, 2004; Gäde, 2004; Isaac et al., 2004; Simonet et al., 2004; Claeys et al., 2005a; Coast and Garside, 2005; Ewer, 2005; Predel and Wegener, 2006; Mertens et al., 2007; Stay and Tobe, 2007; De Loof, 2008; Audsley and Weaver, 2009; Scherkenbeck and Zdobinsky, 2009; Verleyen et al., 2009; Verlinden et al., 2009; Weaver and Audsley, 2009; Altstein and Nässel, 2010; Bendena, 2010; Nässel and Winther, 2010; Van Hiel et al., 2010; Van Loy et al., 2010; Nässel and Wegener, 2011; Herrero, 2012; Spit et al., 2012; Taghert and Nitabach, 2012).

Neuropeptides exert their physiological functions by interacting with specific signal-transducing membrane receptors, resulting in intracellular responses (Zupanc, 1996). Most of these neuropeptide receptors belong to the G protein-coupled receptors (GPCRs), the largest family of cell surface proteins. However, there are some exceptions like the prothoracicotropic hormone (PTTH), which executes its role in metamorphosis through the activation of a receptor tyrosine kinase (RTK) (Rewitz et al., 2009). Most of the insulin-like peptides (ILPs) also interact with RTKs (Fernandez et al., 1995; Graf et al., 1997; Brogiolo et al., 2001; Wheeler et al., 2006; Wen et al., 2010; Iga and Smagghe, 2011). The eclosion hormone (EH), involved in ecdysis, interacts with a membrane-bound guanylate cyclase receptor (Chang et al., 2009) as does the neuropeptide-like precursor peptide 1 (NPLP1) (Overend et al., 2012).

The functional characterization of the first insect neuropeptide receptor, the *Drosophila melanogaster* tachykinin-like receptor (DTKR) took place in 1991 (Li et al., 1991). Subsequently, another *Drosophila* tachykinin-like receptor (NKD) (Monnier et al., 1992) and a neuropeptide Y (NPY)-like receptor (Li et al., 1992) were identified. The latter has recently been deorphanized as the *Drosophila* RYamide receptor (Collin et al., 2011; Ida et al., 2011a). In the following years only a few more insect GPCRs were cloned, e.g., the diuretic hormone receptors of *Manduca sexta* and *Acheta domesticus* (Reagan, 1994, 1996), the *Drosophila* gonadotropin-releasing hormone receptor (Hauser et al., 1998), which later on was deorphanized as an adipokinetic hormone (AKH) receptor (Staubli et al., 2002) and the *Drosophila* allatostatin (AST) receptor (DAR-1) (Birgül et al., 1999).

The real breakthrough in the field of insect neuropeptide receptor research came with the publication of the *Drosophila* genome in 2000 (Adams et al., 2000). This opened the opportunity to predict receptors based on genomic data (Hewes and Taghert, 2001), which clearly boosted the receptor deorphanization rate. At present, 35 GPCRs are functionally characterized in *Drosophila*. One receptor (Dmel\SPR) is activated by seemingly different neuropeptides, the myoinhibitory peptide (MIP) and the sex peptide SP. The others mainly respond to one neuropeptide type, which underlines the specificity of the receptor/ligand couples. Another 14 GPCRs are predicted to be involved in neuropeptide signaling pathways, but their ligands are still unknown and therefore they are classified as “orphan” receptors (Table 1) (Meeusen et al., 2003; Hauser et al., 2006, 2008; Clynen et al., 2010a). In section “Methuselah (CG6936) and Methuselah-like Receptors” the methuselah receptor is also briefly discussed. In spite the fact that several studies have been performed on this receptor, it still is not clear if it is really a neuropeptide receptor and if the *stunted* gene really encodes for its endogenous ligands.

**Table 1:** **Characterized *Drosophila* neuropeptide receptors—Neuropeptide receptors not present in *Drosophila*—Orphan *Drosphila* neuropeptide receptors**.

	**Symbol**	**Receptor gene**	**Endogenous ligand**	**Ligand gene**	Reference^*^
***Drosophila melanogaster* receptor**					
Adipokinetic hormone receptor	Dmel\GRHR	CG11325	Adipokinetic hormone	CG1171	Staubli et al., 2002
Allatostatin A receptor 1	Dmel\AlstR	CG2872	Allatostatin A	CG13633	Larsen et al., 2001
Allatostatin A receptor 2	Dmel\AR-2	CG10001	Allatostatin A	CG13633	Larsen et al., 2001
Allatostatin C receptor 1	Dmel\star1	CG7285	Allatostatin C	CG14919	Kreienkamp et al., 2002
Allatostatin C receptor 2	Dmel\AlCR2	CG13702	Allatostatin C	CG14919	Kreienkamp et al., 2002
Calcitonin-like diuretic hormone receptor	Dmel\Dh31-R1	CG32843/CG17415/CG17043	Diuretic hormone 31	CG13094	Johnson et al., 2005
CAPA receptor	Dmel\capaR	CG14575	Capa-1/Capa-2	CG15520	Iversen et al., 2002a
CCHamide-1 receptor	Dmel\CCHa1r	CG30106/CG14484	CCHamide-1	CG14358	Hansen et al., 2011
CCHamide-2 receptor	Dmel\CCHa2r	CG14593	CCHamide-2	CG14375	Hansen et al., 2011
Cholecystokinin (CCK)-like receptor	Dmel\CCKLR-17D1	CG42301/CG6857	Drosulfakinin	CG18090	Chen et al., 2012
Corazonin receptor	Dmel\GRHRII	CG10698	Corazonin	CG3302	Cazzamali et al., 2002
CRF-like diuretic hormone receptor 1	Dmel\Dh44-R1	CG8422	Diuretic hormone 44	CG8348	Johnson et al., 2004
CRF-like diuretic hormone receptor 2	Dmel\Dh44-R2	CG12370	Diuretic hormone 44	CG8348	Hector et al., 2009
Crustacean cardioactive peptide receptor	Dmel\CcapR	CG33344/CG6111/CG14547	Cardioacceleratory peptide	CG4910	Cazzamali et al., 2003
Ecdysis triggering hormone receptor	Dmel\ETHR	CG5911	Ecdysis triggering hormone	CG18105	Iversen et al., 2002b
FMRFamide receptor	Dmel\FR	CG2114	FMRFamide	CG2346	Cazzamali and Grimmelikhuijzen, 2002
Glycoprotein A2/Glycoprotein B5 receptor	Dmel\Lgr1	CG7665	GPA2/GPB5	CG17878/CG40041	Sudo et al., 2005
Kinin receptor	Dmel\Lkr	CG10626	Leucokinin	CG13480	Radford et al., 2002
Myosuppressin receptor 1	Dmel\DmsR-1	CG8985	Dromyosuppressin	CG6440	Egerod et al., 2003a
Myosuppressin receptor 2	Dmel\DmsR-2	CG43745/CG13803	Dromyosuppressin	CG6440	Egerod et al., 2003a
Neuropeptide F receptor	Dmel\NPFR1	CG1147	Neuropeptide F	CG10342	Garczynski et al., 2002
Pigment dispersing factor receptor	Dmel\Pdfr	CG13758	Pigment-dispersing factor	CG6496	Hyun et al., 2005; Mertens et al., 2005
Proctolin receptor	Dmel\Proc-R	CG6986	Proctolin	CG7105	Johnson et al., 2003a
Pyrokinin 1 receptor	Dmel\Pk1r	CG9918	Drm-PK-1	CG15520	Cazzamali et al., 2005
Pyrokinin 2 receptor	Dmel\CG8784	CG8784	Drm-PK-2	CG6371	Rosenkilde et al., 2003
Pyrokinin 2 receptor	Dmel\CG8795	CG8795	Drm-PK-2	CG6371	Rosenkilde et al., 2003
Rickets	Dmel\rk	CG8930	Bursicon/Partner of burs	CG13419/CG15284	Luo et al., 2005
RYamide receptor	Dmel\NepYr	CG5811	RYamide	CG40733	Ida et al., 2011a
Sex peptide receptor	Dmel\SPR	CG16752/CG12731	Sex peptides^**^ and myoinhibiting peptide precursor	CG17673/CG33495 and CG6456	Yapici et al., 2008; Kim et al., 2010
Short neuropeptide F receptor	Dmel\sNPF-R	CG7395/CG18639	Short neuropeptide F	CG13968	Mertens et al., 2002
SIFamide receptor	Dmel\SIFR	CG10823	SIFamide	CG33527	Jørgensen et al., 2006
Sulfakinin receptor	Dmel\CCKLR-17D3	CG32540/CG6894/CG6881	Drosulfakinin	CG18090	Kubiak et al., 2002
Tachykinin receptor	Dmel\Takr86C	CG6515	Tachykinin	CG14734	Poels et al., 2009
Tachykinin receptor	Dmel\Takr99D	CG7887	Tachykinin	CG14734	Birse et al., 2006
Trissin receptor	Dmel\TrissinR	CG34381/CG14003	Trissin	CG14871	Ida et al., 2011b
**Receptors not present in *Drosophila***					
AKH/corazonin-related peptide receptor	GPRGNR3	XP_321591	ACP	3290616	Hansen et al., 2010
Allatotropin receptor	NGR-A16	NP_001127714	Allatotropin	692738	Yamanaka et al., 2008
Inotocin receptor	ITR	NP_001078830	Inotocin	100038343	Stafflinger et al., 2008
**Orphan *Drosophila* receptors**					
Hector	Dmel\hec	CG4395			
Lgr3	Dmel\Lgr3	CG31096/CG5042			
Lgr4	Dmel\CG34411	CG34411/CG4187			
Moody receptor	Dmel\moody	CG4322			
Trapped in endoderm 1	Dmel\Tre1	CG3171			
Orphan receptor	Dmel\CG4313	CG4313			
Orphan receptor	Dmel\CG12290	CG12290			
Orphan receptor	Dmel\CG32547	CG32547/CG12610			
Orphan receptor	Dmel\CG13229	CG13229			
Orphan receptor	Dmel\CG13995	CG13995			
Orphan receptor	Dmel\CG33696	CG33696/CG16726			
Orphan receptor	Dmel\CG33639	CG33639/CG5936			
Orphan receptor	Dmel\CG30340	CG30340			
Orphan receptor	Dmel\CG13575	CG13575			

* The first published paper in which the full coding cDNA of the receptor was transfected and deorphanized using a reverse pharmacology approach resulting in a physiological relevant EC_50_ value.

** Accessory gland peptide 70A (Acp70A)/Ductus ejaculatorius peptide 99B (Dup99B).

Despite the diversity in their endogenous ligands, GPCRs have been rather well conserved during evolution. This has facilitated the search for neuropeptide receptors in newly released genomes like those of *Apis mellifera* (Hauser et al., 2006), *Tribolium castaneum* (Hauser et al., 2008), and *Bombyx mori* (Yamanaka et al., 2008; Fan et al., 2010). Research in other insects also revealed a set of new neuropeptide signaling systems that are not present in *Drosophila*, e.g., AKH/corazonin-related peptide (ACP) discovered in *Anopheles gambiae* (Hansen et al., 2010), allatotropin (AT) discovered in *B. mori* (Yamanaka et al., 2008), and inotocin discoverd in *T. castaneum* (Stafflinger et al., 2008) (Table 1).

Hitherto, 149 insect genome projects are either completed or in progress (http://www.ncbi.nlm.nih.gov/sites/entrez?db=bioproject) and in 2011, the i5K project was initiated, which aims to sequence 5000 insect genomes in the next 5 years (Robinson et al., 2011). With this overload of genomic information coming up, we intend to give the reader a clear overview of what is currently known on insect neuropeptide receptors. First, we will discuss some general characteristics of GPCRs and the deorphanizing strategies. Next, we will highlight the area of peptidomics, which facilitated the prediction and detection of ligands enormously, followed by a genetics part to discuss some commonly used tools to unravel the physiological functions of the neuropeptide-receptor systems. Thereafter, the current status of the insect neuropeptide GPCRs will be reviewed. To conclude, a short discussion about the importance of neuropeptide research in insects will be given.

## G protein-coupled receptors

Several GPCR-(sub)families originated prior to the divergence of protostomian and deuterostomian animals. This led to a great diversification in chemical specificity to external stimuli like neuropeptides, glycoproteins, nucleotides, biogenic amines, odorants, taste ligands, and photons. Although GPCRs do not share any overall sequence homology, they do expose a similar topographical structure which is remarkably well conserved during evolution. They are typically composed of seven transmembrane (7TM) α-helices, each consisting of 20–30 hydrophobic amino acids, and three extracellular and intracellular loops connecting the different helices. The N-terminus is located at the extracellular site and often possesses several glycosylation sites; the C-terminus, on the other hand, is orientated into the cytoplasm and offers some potential phosphorylation sites. The extracellular parts are involved in ligand-specific binding, while the intracellular areas interact with a member of the family of heterotrimeric GTP-binding proteins (G proteins), consisting of an α-, β-, and γ-subunit (Bockaert and Pin, 1999). Based on shared sequence motifs, the GPCRs are categorized into at least six subfamilies. The evolutionary relationship between the different families is still unclear because of the lack of significant sequence homology. They probably evolved independently of each other or have adopted the G protein signal transduction pathways through convergent evolution (Brody and Cravchik, 2000; Gether, 2000; Horn et al., 2000). All the neuropeptide GPCRs belong to the rhodopsin-like (family A) or the secretin-like (family B) subfamily.

When a GPCR becomes activated by its ligand, the extracellular signal will be transduced into intracellular physiological responses. An activated receptor will undergo a conformational change, which in turn leads to the activation of the associated G protein. This promotes the release of GDP from the α-subunit, followed by binding of GTP. Next, the GTP-bound α-subunit dissociates from the β γ-dimer and both will be released in the cytoplasm. Subsequently, they can interact with their specific effector proteins to elicit cellular signaling pathways. The effector proteins involved depend on the type of the α-subunit. The most common α-subunits are G_q_, G_s_, and G_i/o_. The G_q_ subunits interact with phospholipase Cβ (PLCβ) in order to initiate the hydrolysis of the membrane-bound phosphoinositol-biphospholipid-bisphosphates resulting in diacylglycerol (DAG) and inositol triphosphate (IP_3_). DAG activates protein kinase C (PKC) and IP_3_ mobilizes Ca^2+^ from intracellular stores like the endoplasmic reticulum. The G_s_ and G_i/o_ subunits, respectively, activate or inhibit adenylyl cyclase provoking a subsequent increase or decrease of the cyclic adenosine monophosphate (cAMP) concentration within the cell. The G_s_ proteins are also capable of activating Ca^2+^ channels, while the G_i/o_ proteins are able to interact with K^+^-channels. The intrinsic GTPase activity of Gα induces the hydrolysis of GTP to GDP, resulting in the reassociation of the subunits (Hepler and Gilman, 1992; Lustig et al., 1993; Vanden Broeck, 1996, 2001; Brody and Cravchik, 2000).

## Deorphanizing strategies

There is a clear distinction between the techniques used to deorphanize receptors before and after the genomic era. In the past, one started with a bioactive ligand, purified from tissue extracts, in order to identify its corresponding receptor (the classic approach). Nowadays, an orphan receptor is used to explore its activating ligand from a library of synthetic compounds consisting of predicted neuropeptides (reverse pharmacology) (Meeusen et al., 2003). This strategy makes use of appropriate cellular expression systems used to express orphan receptors of interest. These systems hold the opportunity to measure one of the many second messenger reporter molecules released after receptor activation. The most commonly used expression systems are mammalian cell lines (Chinese Hamster Ovary [CHO] cells or Human Embryonic Kidney [HEK] 293 cells) and *Xenopus* oocytes. These are used in the bioluminescence-based assay (CHO cells), the fluorescence-based assay (HEK293 cells), the luciferase-based assay (HEK293 cells) and the electrophysiological assay (*Xenopus* oocytes).

Because it is nearly impossible to predict which kind of G protein interacts with an orphan receptor, a universal tool was required to predict the signaling cascade. This problem was circumvented with the discovery of the promiscuous G protein α subunits Gα_16_ (human) and Gα_15_ (murine). These Gα proteins regulate PLCβ, and possess the ability to interact with most GPCRs and, as such, their signaling pathways are redirected toward the release of Ca^2+^ (Offermanns and Simon, 1995). Both, the bioluminescence and the fluorescence assay are based on the measurement of the release of intracellular Ca^2+^ upon receptor activation. The bioluminescence assay makes use of bioluminescent proteins such as aequorine, purified from the jellyfish, *Aequoria victoria*, that interact with Ca^2+^ (Prasher et al., 1987; Stables et al., 1997). In the fluorescence assay usually HEK293 cells are charged with a Ca^2+^ sensitive fluorophore that serves as readout (Bender et al., 2002). The luciferase assay makes use of a reporter gene plasmid consisting of a cAMP response element (CRE) as readout for measuring intracellular cAMP levels (Janssen et al., 2008; Horodyski et al., 2011; Vuerinckx et al., 2011). For the electrophysiological assay, *Xenopus* oocytes are injected with a mix of the orphan receptor and the G protein gated inwardly rectifying K^+^ (GIRK) channels that are activated upon ligand binding. This leads to subsequent inward K^+^ currents that can be measured (Kofuji et al., 1995; Ho and Murrell-Lagnado, 1999; Ulens et al., 1999).

Besides the use of these heterologous expression systems, one can also make use of a homologous expression system in which the orphan receptor is expressed in *Drosophila* Schneider-2 (S2) cells (Vanden Broeck et al., 1998). The use of heterologous expression systems, however, prevents that compounds present in an insect extract, or predicted insect ligands would activate endogenous mammalian or amphibian receptors (for reviews, see: Meeusen et al., 2003; Mertens et al., 2004; Beets et al., 2011; Bendena et al., 2012).

## Neuropeptides and peptidomics

An important feature of the currently used deorphanizing strategies is the ability to screen orphan receptors with compound libraries containing potential neuropeptides. The possibility to create such libraries coincided with the availability of the first whole genome databases. This also launched the era of peptidomics, which encloses the purpose to simultaneously identify and/or visualize all peptides present in a cell, tissue, body liquid, or organism. Peptidomics studies are based on two major elements, the *in silico* prediction of neuropeptides and the discovery and identification of neuropeptides using mass spectrometric devices (Baggerman et al., 2002; Predel et al., 2004; Wegener et al., 2006).

Endogenous neuropeptides are enclosed in larger preprohormones, mostly between 50 and 500 amino acids long (Baggerman et al., 2005a). They can code for multiple structurally related or unrelated neuropeptides, as well as for just one neuropeptide. The only common feature of preprohormones is the presence of an amino-terminal signal peptide, with exception of a predicted AST CC neuropeptide in *Drosophila* which has an amino-terminal peptide anchor (Veenstra, 2009a). This peptide is immediately cleaved off after arrival in the endoplasmic reticulum. The residual prohormone undergoes enzymatic cleavage at mono- or dibasic amino acid residues to release the neuropeptides (Hook et al., 2008; Rholam and Fahy, 2009). Most neuropeptides require post-translational modifications to become bioactive or to improve stability.

Because of the poor sequence conservation between preprohormones and the short length of the neuropeptides, the majority consists only of 4–20 amino acids, their prediction from genome databases is not straightforward. Nevertheless, classical BLAST analyses have revealed 36 neuropeptide genes in *D. melanogaster* (Hewes and Taghert, 2001; Vanden Broeck, 2001), and 35 in *A. gambiae* (Riehle et al., 2002). Later on, the combined use of different bioinformatic tools, to overcome the low sensitivity of a BLAST analysis alone, revealed a total of 119 potential neuropeptide-coding genes in *Drosophila* (Liu et al., 2006; Clynen et al., 2010a). All neuropeptides predicted by these methods can be synthesized to construct synthetic peptide libraries applied in the reverse pharmacology assays.

The bioinformatic predictions, though, do not reveal which neuropeptides are ultimately produced, and endogenous bioactive neuropeptides may be overlooked in the genomic data. The processing of a precursor can also differ during developmental stages or between tissues, and post-translational modifications are hard to predict based on sequence information. Therefore, a biochemical characterization of neuropeptides is necessary. There are several possible peptidomics methods available to provide in these needs, all based on mass spectrometry. The most common tool is a combination of liquid chromatography, tandem mass spectrometry and database mining, which allows the detection and sequencing of low concentrations of neuropeptides from complex mixtures (Clynen et al., 2010b). Mass spectrometry applications led to the discovery of hundreds of neuropeptides. As is often the case, *Drosophila* peptidomics (Baggerman et al., 2002, 2005b; Schoofs and Baggerman, 2003) paved the way for peptidomic studies in other insects, e.g., *A. mellifera* (Hummon et al., 2006; Boerjan et al., 2010a), *Nasonia vitripennis* (Hauser et al., 2010), *T. castaneum* (Li et al., 2008), and *Aedes aegypti* (Predel et al., 2010). Also in insects with no completely sequenced genome, peptidomics may prove useful, e.g., *Locusta migratoria* (Clynen et al., 2006; for reviews, see: Hummon et al., 2006; Boonen et al., 2008; Menschaert et al., 2010).

## Functional genomics

Upon the characterization of a neuropeptide receptor and its ligand, the question remains which function they possess in a specific organism. These functions can be determined with genetic tools. In the classic approach the phenotype of interest is chosen first and then attempts are made to identify the genes responsible for this phenotype (forward genetics). With the rise of the whole genome era, a tremendous number of genes with unknown functions were identified. This made it possible to start with a gene of interest and to study its function (reverse genetics). Currently, the most used techniques to perform reverse genetics are silencing of genes of interest by RNA interference (RNAi), generating knockouts, and overexpressing specific genes using the GAL4/UAS system.

The generation of loss-of-function phenotypes through the application of RNAi is a fairly new technique as it was described for the first time in 1998 in *Caenorhabditis elegans* (Fire et al., 1998), immediately followed by a report of RNAi usage in *D. melanogaster* (Kennerdell and Carthew, 1998). RNAi studies are widely used in the field of insect research and have proven to be appropriate to unravel functions of neuropeptides and their receptors in various species (Bellés, 2010; An et al., 2012). There is a genome-wide transgenic RNAi library available for *Drosophila*, consisting of short gene fragments cloned as inverted repeats and expressed using the binary GAL4/UAS system (Dietzl et al., 2007). The usage of the GAL4/UAS system to perform RNAi experiments becomes also more established in other insects like *B. mori* (Dai et al., 2008) and *T. castaneum* (Schinko et al., 2010).

RNAi can not entirely impede the expression of a gene of interest. To generate a complete knockout of a gene, mutagenic or homologous recombination tools are frequently used. Mutagenesis relies on the incorporation of mutations, which can be obtained by the application of chemical mutagenesis or by transposable element mutagenesis, followed by a thorough screen to detect the samples containing mutations in the gene of interest. Homologous recombination is based on the host DNA repair system for the alteration of a target sequence in the genome by a donor sequence. This donor sequence exhibits homology to the target sequence, but contains the desired genetic modifications. The alteration is preceded by the generation of a double strand break in the target or donor sequence, inducing the homologous recombination repair system (for reviews, see: Reumer et al., 2008; Wesolowska and Rong, 2010; An et al., 2012).

Besides studying the effects of a knockdown or a complete knockout of a certain gene, overexpressing a gene can also yield important information about its function. To obtain overexpression, the gene of interest can be coupled to a binary GAL4/UAS system as well.

The previous described techniques to identify, deorphanize and determine the functions of neuropeptide signaling systems are widely applied in insect research, yielding an enormous amount of information. Table 2 summarizes the neuropeptide receptors that have been predicted and/or functionally characterized for a selection of model insects. In the next section we aim to give a brief summary of what is known so far relating to these insect neuropeptide receptors. For convenience all intertitles are accompanied with the corresponding computed gene (CG) numbers of the *Drosophila* receptors. These numbers were used for genes identified during the annotation of the whole *Drosophila* genome sequence. For those receptor genes not annotated in *Drosophila*, the accession number of the receptor gene for the insect in which it was first deorphanized is added.

**Table 2:**
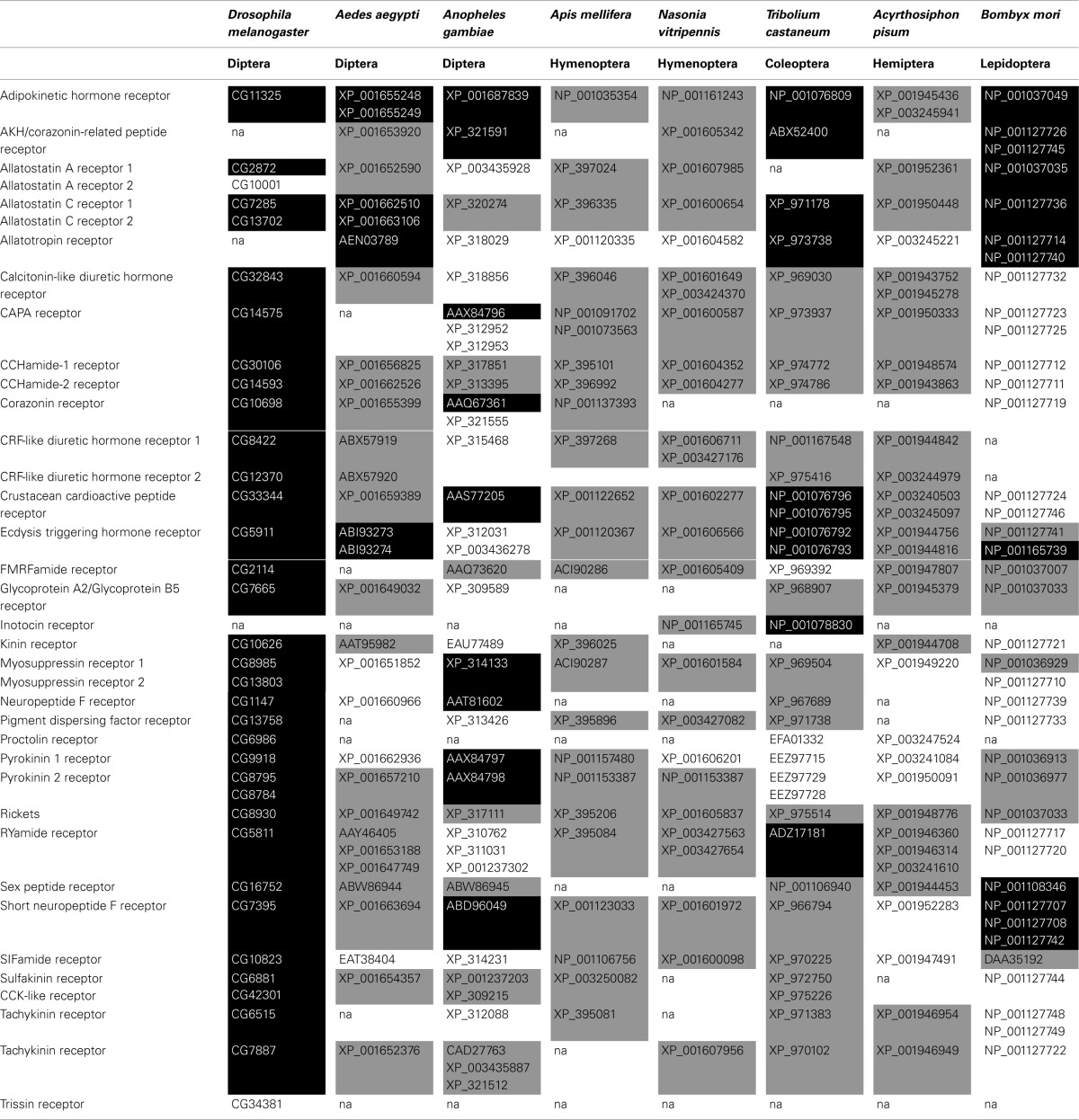
**Characterized and predicted neuropeptide receptors in insect species of varying insect orders**.

Black box: functionally characterized neuropeptide receptors.

Gray box: previously predicted neuropeptide receptors.

White box: predicted neuropeptide receptors based on our own blast analysis.

na: not annotated.

## Deorphanized neuropeptide receptors

### Adipokinetic hormone receptors (CG11325 Orthologs)

The first structural characterization of an AKH neuropeptide was achieved in 1976 (Stone et al., 1976). Currently, around 55 isoforms, derived from various insect species, have been described (Gäde, 2009; Caers et al., 2012; Gäde and Marco, 2012; Jedlička et al., 2012; Malik et al., 2012; Weaver et al., 2012). They consist of 8–10 amino acids, and are characterized by a blocked N-terminus (pyroglutamate) and C-terminus (amidation) (Gäde and Auerswald, 2003). The main function of AKH is the regulation of the energy metabolism. During energy requiring processes like flight, the AKH neuropeptides are released from the corpora cardiac (CC) and will interact with their receptors, present in the membrane of the fat body adipocytes. This will induce the release of energy rich substrates (lipids, trehalose, or proline) (Lorenz and Gäde, 2009). The kind of substrates released, depends on the coupled G protein. When AKH binds to a G_q_ protein-coupled receptor, glycogen phosphorylase will be activated and trehalose will be set free. If the signaling pathway acts by a G_s_ protein-coupled receptor, triacylglycerol lipase will be activated, resulting in the production of DAG or free fatty acids (Gäde and Auerswald, 2003). The last years it became clear that the function of AKH is not restricted to locomotory activity alone, but that it acts as a general regulator of homeostasis in insects, influencing all energy requiring processes (e.g., egg production, feeding behavior, larval growth, molting, and immune response) (Goldsworthy et al., 2002, 2003; Lorenz, 2003; Lee and Park, 2004; Isabel et al., 2005; Grönke et al., 2007; Bharucha et al., 2008; Lorenz and Gäde, 2009; Arrese and Soulages, 2010; Attardo et al., 2012; Konuma et al., 2012). AKH also serves as an anti-stress hormone in oxidative stress situations (Kodrík et al., 2007; Večeřa et al., 2007; Kodrík, 2008; Huang et al., 2011a).

The AKH receptors (AKHR) are closely related to the ACP receptors and constitute the invertebrate AKH/ACP receptor family. Together with the invertebrate corazonin/gonadotropin releasing hormone (GnRH) receptor family and the vertebrate/protochordate GnRH receptor family they compose the GnRH receptor superfamily (Lindemans et al., 2011; Roch et al., 2011). The first AKHR was determined in *M. sexta* by using fat body fractions to ascertain the optimal binding conditions for tritium-labeled *Manse*-AKH (Ziegler et al., 1995). The *Drosophila* AKHR was the first to be cloned and was deorphanized by making use of the electrophysiological assay (Park et al., 2002), and its characterization was confirmed by Staubli et al. (2002) using a bioluminescence assay. Later, AKHRs were also identified and characterized in other insect species: *Periplaneta americana* (Hansen et al., 2006; Wicher et al., 2006a), *A. gambiae* (Belmont et al., 2006), *B. mori* (Staubli et al., 2002; Zhu et al., 2009; Huang et al., 2010), and *T. castaneum* (Li, unpublished data). Two putative AKHR variants have been predicted in *A. aegypti* (Kaufmann et al., 2009) and one AKHR is identified in the *Apis* genome (Hauser et al., 2006). However, it remains doubtful if this receptor is really functional in the honeybee, because mass spectrometric techniques have failed to detect the predicted *Apis* AKH neuropeptide (Veenstra et al., 2012). Besides expression in the fat body (Kaufmann and Brown, 2006; Ziegler et al., 2011), the AKHR is also expressed in various neurons of *P. americana*, including the abdominal dorsal unpaired medial (DUM) neurons, which are responsible for the release of octopamine. As such, octopamine may be the link between elevated AKH-titers and the increase in locomotion (Wicher et al., 2006b, 2007; Verlinden et al., 2010).

### Adipokinetic hormone/corazonin-related peptide receptor (XP_321591 orthologs)

In 2006, an *A. gambiae* receptor was annotated and cloned that was closely related to the AKH and corazonin receptors, but could not be activated by these neuropeptides (Belmont et al., 2006). Hansen et al. (2010) detected a neuropeptide closely related to both AKH and corazonin and named it ACP. This neuropeptide was able to activate the receptor expressed in CHO/Gα_16_ cells in a dose-responsive manner (Hansen et al., 2010). Subsequently, the ACP receptor was also characterized in *T. castaneum* (Hansen et al., 2010). Recently, two predicted ACP receptors of *B. mori* (Yamanaka et al., 2008; Hansen et al., 2010) were also characterized, but were indicated as AKHR (Shi et al., 2011). The ACP neuropeptides were in fact already described in *L. migratoria* (Siegert, 1999) and in *A. gambiae* (Kaufmann and Brown, 2006), but were classified as AKH neuropeptides with unknown functions. ACP and its receptor are structurally intermediate between the AKH and corazonin neuropeptides and their receptors, which is a prominent example of receptor/ligand co-evolution. An ancestral receptor and ligand gene have probably duplicated several times followed by mutations and evolutionary selection, leading to three signaling systems. However, the ACP signaling system is absent in all investigated *Drosophila* species as well as in *A. mellifera*, *Acyrthosiphon pisum*, *Pediculus humanus*, and in the crustacean *Daphnia pulex*, suggesting that it may have been lost several times during arthropod evolution (Hansen et al., 2010). So far no functions are assigned to the ACP signaling system, but the high expression shortly before and after hatching of *T. castaneum* suggests a role in early larval development (Hansen et al., 2010).

### Allatostatin a receptors (CG2872 and CG10001 orthologs)

The endogenous ligands of the AST A receptor are the A-type AST neuropeptides which belong to the group of allatoregulatory neuropeptides together with the B-, and C-type allatostins and the ATs (for a review, see: Weaver and Audsley, 2009) and the recently discovered AST CC neuropeptides (Veenstra, 2009a). Allatoregulatory peptides are named after their ability to either inhibit (ASTs) or stimulate (ATs) juvenile hormone (JH) synthesis (Audsley et al., 2008). The B-type ASTs are also known as myoinhibiting peptides and were found to activate the SP receptor [see section “Sex Peptide/Myoinhibiting Peptide Receptor (CG16752/CG12731 Orthologs)”].

The A-type AST-As, or FGLamides were first isolated of brain extracts of cockroaches (Woodhead et al., 1989; Pratt et al., 1991), and have since been found in every investigated insect species, except for *T. castaneum* (Li et al., 2008). They are characterized by a conserved (Y/F)XFG(L/I)-NH_2_ sequence (Hayes et al., 1994; Audsley et al., 1998). AST-As regulate JH biosynthesis in cockroaches, crickets, and termites (Pratt et al., 1989, 1991; Woodhead et al., 1989, 1994; Bellés et al., 1994; Weaver et al., 1994; Lorenz et al., 1995, 1999; Yagi et al., 2005; for a review, see: Stay and Tobe, 2007). A property attributed to all AST-As is myoinhibition of visceral muscles (Hoffmann et al., 1999; Stay, 2000; Aguilar et al., 2003; Weaver and Audsley, 2009; Zandawala et al., 2012). Recently, *Drosophila* AST-A was linked to food intake and foraging behavior (Hergarden et al., 2012; Wang et al., 2012). In *Drosophila*, two A-type AST receptors are identified: DAR-1 and DAR-2 (Birgül et al., 1999; Larsen et al., 2001). DAR-1, when expressed in *Xenopus* oocytes was shown to couple to a G-protein of the G_i/o_ family. When expressed in CHO cells, DAR-1 and -2 are activated by AST-A and mobilize intracellular Ca^2+^ (Larsen et al., 2001). AST-A receptors were also characterized in *P. americana* (Auerswald et al., 2001), *B. mori* (Secher et al., 2001), and *Diploptera punctata* (Lungchukiet et al., 2008). Northern blot experiments showed that the *B. mori* receptor is expressed in the midgut of fifth larval instars and to a much lesser extend in the brain (Secher et al., 2001).

### Allatostatin C receptors (CG7285 and CG13702 orthologs)

The first C- or PISCF-type AST was characterized in the late pupae of *M. sexta*. AST-Cs contain a typical C-terminal PISCF-OH sequence, a blocked N-terminus and a disulfide bridge linking Cys-7 and Cys-14 (Kramer et al., 1991). Orthologs are found in other lepidopteran, dipteran and coleopteran species (Li et al., 2006). In several insects, C-type or C-type-like ASTs can have both allatostatic and allatotropic properties, depending on the age of the animal (Abdel-Latief et al., 2004; Clark et al., 2008; Griebler et al., 2008; Abdel-Latief and Hoffmann, 2010). In Diptera two AST-C receptors have been characterized for *Drosophila* using *Xenopus* oocytes (Kreienkamp et al., 2002) and for *Aedes* using HEK cells (Mayoral et al., 2010). Only one AST-C receptor was found to be present in *Bombyx* (Yamanaka et al., 2008) and in *Tribolium* (Audsley et al., 2012). Activation of the *Bombyx* AST-C receptor elicits an increase in intracellular cAMP levels (Yamanaka et al., 2008), while the *Tribolium* receptor was deorphanized in HEK cells, inducing a Ca^2+^ response (Audsley et al., 2012). In adult fruit flies, both *drostar* genes are expressed in the optic lobes and the pars intercerebralis, where the AST-C neuropeptide was also found to be present. This suggests a function in the modulation of visual information processing. In the last larval stage, receptor expression was found in the brain and corpora allata (CA) (Kreienkamp et al., 2002). In *Aedes* significant differences were observed in tissue distribution and expression levels for the two receptor paralogs (Mayoral et al., 2010). In *Tribolium* the highest transcript levels were noticed in the head and the gut, with variable amounts in the fat body and reproductive organs. These transcript levels were also shown to be sex-dependent (Audsley et al., 2012).

The recently discovered AST CC neuropeptide (AST CC) (Veenstra, 2009a) was also identified in *Tribolium* and showed to be capable of activating the AST-C receptor in a dose-dependent manner (Audsley et al., 2012). A knock out of the *Drosophila Ast-CC* gene is embryonic lethal, suggesting that it is an essential gene (Veenstra, 2009a).

### Allatotropin receptors (NP_001127714 Orthologs)

AT was named after its ability to stimulate JH biosynthesis in the CA but is also linked to other functions like myostimulation, cardio-acceleration, regulation of photic entrainment, ion exchange regulation, and the up-regulation of the secretion of digestive enzymes (Veenstra et al., 1994; Würden and Homberg, 1995; Lee et al., 1998; Koladich et al., 2002; Petri et al., 2002; Homberg et al., 2003; Hofer and Homberg, 2006; Lwalaba et al., 2010; Sterkel et al., 2010), of which the myotropic role of AT is probably the most ancestral (Elekonich and Horodyski, 2003). ATs are found in several invertebrate EST and genomic databases (for reviews, see: Clynen and Schoofs, 2009; Weaver and Audsley, 2009) and they all have a TARGF/Y motif at the C-terminus. In *Manduca* and *Bombyx*, also AT-like (ATL) neuropeptides were found, which arise by alternative splicing of the AT gene (Horodyski et al., 2001; Nagata et al., 2012a). In 2008, the AT receptor (ATR) was characterized in *B. mori* (Yamanaka et al., 2008). Remarkable, this receptor was mainly localized in the Short neuropeptide F (sNPF)-producing cells in the CC, but not in the JH producing CA. It was suggested that AT regulates the production and/or release of sNPFs from the CC and that these sNPFs are responsible for some of the allatotropic functions assigned to the ATs (Yamanaka et al., 2008). In 2011, the ATRs of *M. sexta*, *T. castaneum*, and *A. aegypti* were characterized (Horodyski et al., 2011; Vuerinckx et al., 2011; Nouzova et al., 2012) and show, unlike the ligand, remarkable similarity with the vertebrate orexin receptors (Yamanaka et al., 2008; Vuerinckx et al., 2011). Upon activation by AT or ATLs, *Manse*-ATR, and *Trica*-ATR elevate both intracellular Ca^2+^ and cAMP concentrations in cellular expression systems (Horodyski et al., 2011; Vuerinckx et al., 2011). Expression of ATs and ATRs in the different insect species is likely to be strongly regulated, since large differences were measured between developmental stages, sexes, feeding conditions, *etc*. (Elekonich and Horodyski, 2003; Horodyski et al., 2011; Vuerinckx et al., 2011; Nouzova et al., 2012). Possibly additional ATRs may be present in some insect species, since very similar additional receptors have been predicted from *Bombyx* and *Aedes* genomes (Yamanaka et al., 2008; Nouzova et al., 2012).

### Calcitonin-like diuretic hormone receptors (CG32843/CG17415/CG17043 Orthologs)

The first calcitonin-like diuretic hormone (CT/DH), called *Dippu*-DH_31_ was identified in *D. punctata* (Furuya et al., 2000). More orthologs were discovered by phylogenetic analysis (Zandawala, 2012). CT/DH stimulates fluid secretion by Malpighian tubules and seems to work via a Ca^2+^-dependent mechanism in *D. punctata* (Furuya et al., 2000). In *Drosophila*, CT/DH stimulates fluid secretion by activating the apical membrane V-ATPases via cAMP as second messenger (Coast et al., 2001) and in *Anopheles* the fluid excretion in Malpighian tubules is also cAMP driven (Coast et al., 2005). In *Rhodnius*, diuresis by CT/DH seems to be independent of cAMP (Te Brugge et al., 2011). CT/DH is also involved in contractions of the gut and associated glands (Te Brugge et al., 2009) and may play a role in ecdysis (Kim et al., 2006a,b). The *Drosophila* CT/DH receptor (DH31-R1) is activated by *Drome*-DH_31_ and is expressed in the Malpighian tubules. The signaling in HEK293 cells was dependent upon co-expression of the receptor component protein (RCP), which is critical for downstream signaling from the mammalian calcitonin-like receptor (Johnson et al., 2005). One CT/DH receptor has been predicted in *A. aegypti*, *A. gambiae*, *A. mellifera*, *N. vitripennis*, and *T. castaneum* and two CT/DH receptors were found in *A. pisum*, although it is not yet clear whether both paralogues encode a functional CT/DH receptor.

### Capa receptors (CG14575 Orthologs)

The insect capa neuropeptides, or periviscerokinin peptides, usually possess the C-terminal sequence FPRVamide. The insect *capability* gene encodes a preprohormone containing two capa neuropeptides (capa-1 and capa-2) and one or more pyrokinin-1 (Kean et al., 2002), but they do not activate each other's receptors (Iversen et al., 2002a; Rosenkilde et al., 2003; Cazzamali et al., 2005). Capa neuropeptides have a diuretic effect on the Malpighian tubules of *Drosophila* (Pollock et al., 2004), but in *R. prolixus* and other insects they act antidiuretic (Coast and Garside, 2005; Paluzzi and Orchard, 2006). Recently, it was shown that the *Aedes* capa neuropeptide can induce either diuretic or antidiuretic effects depending on the dose (Ionescu and Donini, 2012). In addition, capa neuropeptides have myotropic effects in a variety of insects (Wegener et al., 2002; Predel and Wegener, 2006). Capa receptors have been characterized in *Drosophila* and in *Anopheles* (Iversen et al., 2002a; Park et al., 2002; Olsen et al., 2007; Terhzaz et al., 2012). Both capa-1 and capa-2 elicited a dose-dependent response. The gene encoding the *Drosophila* capa receptor is highly expressed in larval and adult tubules (Terhzaz et al., 2012).

Capa receptors are found in different mosquito species, although not in *A. aegypti*. In other holometabolous insects, orthologs are found in representatives of the major orders, including Hymenoptera, Coleoptera, and Lepidoptera. The honey bee genome contains two paralogues, as does the *B. mori* and *M. sexta* genome. Also *N. vitripennis* contains a paralogue (XP_001600587.2), formerly suggested lacking this receptor (Hauser et al., 2006, 2010; Yamanaka et al., 2008). Also in Coleoptera, a capa receptor is found in *Tribolium* (Hauser et al., 2008).

### CCHamide-1 and -2 receptors (CG30106/CG14484 and CG14593 Orthologs)

The first CCHamide neuropeptide has only recently been identified in *B. mori* and it was found to be expressed in the central nervous system and the midgut (Roller et al., 2008). Subsequently, two CCHamide neuropeptides were detected in all insects with a sequenced genome (Hansen et al., 2011). In *D. melanogaster*, cognate receptors have been identified for both CCHamide neuropeptides. CG30106 expressed in CHO/Gα_16_ cells was activated by CCHamide-1 at nanomolar concentrations but also responded to high concentrations of CCHamide-2. CG14593 was activated by nanomolar concentrations of CCHamide-2 as well as by micromolar concentrations of CCHamide-1 (Hansen et al., 2011). Previously, CG30106 had been described as a receptor for myoinhibiting neuropeptides (Johnson et al., 2003b), but as several independent attempts to repeat this result were fruitless, this was likely an erroneous characterization.

### Corazonin receptors (CG10698 Orthologs)

The first corazonin was isolated and identified from the CC of *P. americana* and was presented as a new cardioaccelerating neuropeptide (Veenstra, 1989). Corazonin is present in most insects (excluding beetles and aphids) (for reviews, see: Gäde et al., 2008; Li et al., 2008; Weaver and Audsley, 2008; Huybrechts et al., 2010) and the most common corazonin sequence among insects is pQTFQYSRGWTNamide (Predel et al., 2007). The role of corazonin, however, is not restricted to cardio-excitatory actions. In locusts, corazonin is involved in cuticular melanization in the gregarious phase (Tawfik et al., 1999; Tanaka et al., 2002), in *M. sexta* a role in the initiation of ecdysis behavior is noticed (Kim et al., 2004; Žitňan et al., 2007) and it has been suggested that corazonin is involved in sex-dependent stress responses (Zhao et al., 2010) and in the regulation of insulin producing cells in *Drosophila* ((Kapan et al., 2012); for reviews, see: Veenstra, 2009b; Boerjan et al., 2010b).

The corazonin receptor was first characterized in *Drosophila* by making use of a bioluminescence assay (Cazzamali et al., 2002), which was confirmed using *Xenopus* oocytes (Park et al., 2002). Subsequently, the corazonin receptors for *M. sexta* (Kim et al., 2004), *A. gambiae* (Belmont et al., 2006) and *B. mori* (Shi et al., 2011) were characterized, and a putative corazonin receptor for *Musca domestica*, was cloned (Sha et al., 2012). Neither the corazonin neuropeptide nor its receptor could be identified in *Tribolium* (Hauser et al., 2008) or *Acyrthosiphon*. In *N. vitripennis*, despite the presence of a corazonin neuropeptide, so far no corazonin receptor could be predicted (Hauser et al., 2010). The invertebrate corazonin receptors are part of the GnRH receptor superfamily [see section “Adipokinetic Hormone Receptors (CG11325 Orthologs)”] (Lindemans et al., 2011; Roch et al., 2011). The *Drosophila* receptor is expressed in all developmental stages (Cazzamali et al., 2002). The *Manduca* corazonin receptor is present in endocrine Inka cells, the source of preecdysis- and ecdysis-triggering hormones, suggesting a role upstream of ecdysis triggering hormone (ETH) (Kim et al., 2004). In *Anopheles*, there are pronounced spikes of corazonin receptor expression in 2nd instar larvae and around the transition from pupa to adult (Hillyer et al., 2012). In *Musca*, a high level of corazonin receptor expression was noticed in the larval salivary glands and a moderate level in the central nervous system. In adults, the receptor was expressed both in the head and body (Sha et al., 2012).

### CRF-like diuretic hormone receptors (CG8422 and CG12370 Orthologs)

The first corticotropin-releasing factor like diuretic hormone (CRF/DH) was identified in *M. sexta* as a diuretic peptide (DP) consisting of 41 amino acids that shows sequence similarity to corticotropin releasing factor, urotensin I and sauvagine (Kataoka et al., 1989). A second CRF/DH was also discovered in *M. sexta* (Blackburn et al., 1991). CRF/DHs are also referred to as DH_44_, after the number of amino acids in the CRF/DH of *D. melanogaster* (Cabrero et al., 2002). CRF/DH increases fluid excretion *in vivo* (Kataoka et al., 1989) and *in vitro* (Kay et al., 1991, 1992; Lehmberg et al., 1991; Clottens et al., 1994) and increases cAMP levels in Malpighian tubules (Lehmberg et al., 1991; Kay et al., 1992; Clottens et al., 1994; Furuya et al., 1995). Besides its diuretic function, CRF/DH negatively influences feeding and reproduction (Keeley et al., 1992; Van Wielendaele et al., 2012) and stimulates gut contractions (Te Brugge et al., 2009). The *M. sexta* CRF/DH receptor was the first to be cloned and was activated by *Manse*-DH, making use of cAMP as second messenger (Reagan, 1995). Also the CRF/DH receptor in *A. domesticus* uses cAMP as secondary messenger (Reagan, 1996). The first *D. melanogaster* CRF/DH receptor (DH44-R1), encoded by CG8422, may couple to multiple second messengers as both cAMP and Ca^2+^ were stimulated upon binding of *Drome*-DH to the receptor (Johnson et al., 2004). The second *D. melanogaster* CRF/DH receptor (DH44-R2), encoded by CG12370, is also activated by *Drome*-DH resulting in an increase of intracellular cAMP and causes specific β-arrestin translocation to the plasma membrane. DH44-R2 is probably the receptor that modulates DH sensitivity at the level of the microtubules (Hector et al., 2009). A CRF/DH receptor was also cloned in *B. mori* and in *A. aegypti* (Ha et al., 2000). The *Aedes* DH-I receptor is by far the most abundant receptor in Malpighian tubules and its transcript levels increase after a blood meal (Jagge and Pietrantonio, 2008). More CRF/DH receptor orthologs were found in *T. castaneum* and *A. pisum*, but only one orthologue is found in *A. gambiae*, *A. mellifera*, and *N. vitripennis* up to date. Although the number of receptors seems to differ, CRF/DH signaling is likely to be conserved in all major insect orders.

### Crustacean cardioactive peptide receptors (CG33344/CG6111/CG14547 Orthologs)

Crustacean cardioactive peptide (CCAP) was originally identified in the shore crab *Carcinus maenas* and exhibited an acceleratory effect on semi-isolated heart tissue (Stangier et al., 1987). An identical neuropeptide was subsequently isolated from *L. migratoria* (Stangier et al., 1989). The structure of CCAP is identical in all examined insects and consists of the cyclic nonapeptide PFCNAFTGCamide. CCAP stimulates heart contractions (Cheung et al., 1992; Furuya et al., 1993; Li et al., 2011a) and contractions of visceral muscles (Stangier et al., 1989; Donini et al., 2001, 2002; Donini and Lange, 2002), and promotes the release of AKH (Veelaert et al., 1997) and digestive enzymes (Sakai et al., 2006). CCAP also plays a role in ecdysis in several insects (Gammie and Truman, 1997; Ewer et al., 1998; Kim et al., 2006a,b; Arakane et al., 2008). *Drosophila* and *Anopheles* CCAP receptors have been expressed in CHO/Gα_16_ cells and are activated by CCAP (Cazzamali et al., 2003; Belmont et al., 2006). In *T. castaneum*, two genes encode for CCAP receptors (Hauser et al., 2008) and both showed a dose-dependent response to CCAP (Li et al., 2011a). Functional analysis using RNAi revealed that only TcCCAPR-2 is essential for cardioacceleratory activity (Li et al., 2011a). CCAP receptor orthologs have been found in *A. mellifera* (Hauser et al., 2006), *A. aegypti*, *A. pisum*, *B. mori*, and *N. vitripennis* and thus the CCAP receptor seems to be conserved in many insect orders.

### Ecdysis triggering hormone receptors (CG5911 Orthologs)

To be able to grow and undergo metamorphosis, insects need to shed their exoskeleton, the process known as ecdysis (Truman, 1996). This process is initiated and regulated by the ETH (for a review, see: Žitňan et al., 2007). The *eth* gene encodes for two active neuropeptides named pre-ETH and ETH in moths and ETH1 and ETH2 in other insects. The ETHs have a common PRX_1_-amide (X_1_ is I, V, L, or M) sequence at the C-terminus (Park et al., 2002). In *Drosophila*, *Manduca*, and *Bombyx*, the two ETHs differ in length. In *Drosophila* and *Manduca* the short form only can elicit a part of the ecdysis behaviors, whereas the long one can elicit whole ecdysis (Žitňan et al., 1999; Park et al., 2002). In *Bombyx* and *Aedes*, both neuropeptides seemed to be equally potent (Žitňan et al., 2002; Dai and Adams, 2009). In *Apis*, *Nasonia*, and *Acyrthosiphon* only one form is found, that in *Apis* is shown to be sufficient to elicit ecdysis (Žitňan et al., 1999; Park et al., 2002). These neuropeptides are released in the bloodstream and activate the ETH receptors (ETHRs) situated in the central nervous system. The *ethr* gene encodes for two splice variants of the receptor, ETRH-A and ETRH-B (Iversen et al., 2002b; Park et al., 2002; Dai and Adams, 2009; Roller et al., 2010), and the first ETHRs were identified in *Drosophila* (Iversen et al., 2002b; Park et al., 2002). The two forms are expressed in different central neurons (Kim et al., 2006a,b). ETHR-A is expressed in inhibitory and/or excitatory neuropeptide producing neurons, releasing the neuropeptides in response to ETH to regulate ecdysis (Kim et al., 2006a,b). In *B. mori* ETHR-B is highly expressed in the CA, pointing to a possible allatoregulatory function (Yamanaka et al., 2008). In *Drosophila*, *Manduca*, and *Aedes* activation of both receptors expressed in CHO cells could increase intracellular Ca^2+^ levels (Iversen et al., 2002b; Park et al., 2002; Kim et al., 2006a,b; Dai and Adams, 2009). In *Bombyx*, ETHR-B was expressed in HEK293 cells and was shown to be able to increase intracellular cAMP levels (Yamanaka et al., 2008). In *Tribolium*, the function of the ETRHs was confirmed through RNAi experiments (Arakane et al., 2008). ETRHs were also found in several holo- and hemimetabolous insects (Riehle et al., 2002; Žitňan et al., 2003; Clynen et al., 2006; Roller et al., 2010).

### FMRFamide receptors (CG2114 Orthologs)

The family of (N-terminally extended) FMRFamides is named after the tetrapeptide FMRFamide that was identified in the sunray venus clam *Macrocallista nimbosa* (Price and Greenberg, 1977), but not all extended FMRFamides retain the exact C-terminal motif. The first extended FMRFamide in insects was cloned and characterized in *D. melanogaster* (Nambu et al., 1988; Schneider and Taghert, 1988). More extended FMRFamides were detected by mass spectrometric analysis in various major insect orders (Verleyen et al., 2004a; Neupert and Predel, 2005; Li et al., 2008; Ons et al., 2009; Rahman et al., 2009; Huybrechts et al., 2010; Audsley et al., 2011; Zoephel et al., 2012). FMRFamides modulate heart and gut contractions in insects (Banner and Osborne, 1989; Robb and Evans, 1990; Duttlinger et al., 2002). The FMRFamide neurons become active at the early stages of pre-ecdysis in *D. melanogaster*, suggesting a role in the ecdysis process (Kim et al., 2006b). The *Drosophila* FMRFamide receptor is the only deorphanized insect FMRFamide receptor so far and was found to be activated by six of the seven endogenous *D. melanogaster* extended FMRFamides (Cazzamali and Grimmelikhuijzen, 2002; Meeusen et al., 2002). Orthologous FMRFamide receptors are found in *A. gambiae* (Duttlinger et al., 2003), *A. mellifera*, *N. vitripennis*, *T. castaneum*, *A. pisum*, and *B. mori* but have not been characterized up to date. FMRFamide receptors are conserved throughout insects, but our knowledge about these receptors is very limited.

### Inotocin receptor (NP_001078830 Orthologs)

This neuropeptide was first discovered in the 1980s in *L. migratoria* and showed similarity to the oxytocin/vasopressin peptide family in Mammalia. The antiparallel dimer of the neuropeptide was described to have diuretic properties (Proux et al., 1987). Although the neuropeptide could not be identified in most insect species with sequenced genomes, it was recently found in *T. castaneum* and *N. vitripennis*. The mature neuropeptide shows C-terminal amidation. The *T. castaneum* inotocin receptor was characterized in CHO/Gα_16_ cells displaying strong activation in the nanomolar range. For both the neuropeptide precursor and its receptor transcript levels have been reported throughout development of *T. castaneum*, but in larvae and the head of adult beetles high levels were detected (Aikins et al., 2008; Stafflinger et al., 2008). Inotocin was shown to act indirectly as a diuretic factor on *Tenebrio molitor* Malpighian tubules in the presence of central nervous system and CC-CA (Aikins et al., 2008).

### Kinin (myokinin) receptors (CG10626 Orthologs)

Insect kinins are small neuropeptides that function as myotropic, neuromodulatory, and diuretic hormones in the insect Malphigian tubules (Hayes et al., 1989; Terhzaz et al., 1999; Coast and Garside, 2005). These neuropeptides, which are characterized by the C-terminal sequence FX_1_X_2_WGamide (where X_1_ is F, H, N, S or Y and X_2_ is A, P, or S), were first isolated from *Leucophea maderae* (Holman et al., 1987; Hayes et al., 1989). The *Drosophila* kinin receptor was deorphanized in S2 cells using a bioluminescence assay (Radford et al., 2002). Antibodies raised against the receptor identified sites of myokinin action like stellate cells of the Malphigian tubules, two triplets of cells in the pars intercerebralis of the adult central nervous sytem and additional cells in the larval nervous system. Western blots and reverse transcription-PCR confirmed these locations, but also identified expression in male and female gonads. These tissues also displayed elevated Ca^2+^ in response to myokinin, demonstrating novel roles for these neuropeptides (Radford et al., 2002). In *A. aegypti* the myokinin receptor was shown to be critical for *in vivo* fluid excretion post blood feeding (Kersch and Pietrantonio, 2011). In *Drosophila* the receptor was shown to be involved in appetite, chemosensory responses, and metabolism (Al-Anzi et al., 2010; de Haro et al., 2010; Cognigni et al., 2011; López-Arias et al., 2011). Receptor orthologs are also present in *A. mellifera* (Hauser et al., 2006), *A. gambiae*, *Culex quinquefasciatus*, *A. pisum*, *P. humanus*, and *B. mori*, but seem to be absent in *N. vitripennis* and *T. castaneum*.

### Leucine-rich repeats containing GPCRs (LGRs)

These receptors, which belong to the rhodopsin-like GPCRs, can be considered “the odd ones out” within this receptor family as they display ectodomains that are much larger than is generally the case for rhodopsin-like GPCRs. Based on the structure of the ectodomain and the hinge region which links the ectodomain to the serpentine domain, three major types can be identified within the LGR family (Hsu et al., 2000; Van Hiel et al., 2012).

#### Type A LGRs (CG7665 Orthologs)

Type A LGRs typically have 7–9 leucine-rich repeats (LRRs) in their ectodomain. Although little data are available on these receptors in insects, they are thought to be of significant importance as they are homologous to the three vertebrate receptors for the glycoprotein hormones (follicle stimulating hormone, thyroid stimulating hormone, luteinizing hormone, and choriogonadotropin). In contrast to the situation in vertebrates, invertebrate genomes encode only one type A LGR and the receptor is conserved in most sequenced insect genomes, but seems to be lost in Hymenoptera (Hauser et al., 2006, 2010; Fan et al., 2010). Another exception is the *T. castaneum* genome which encodes two type A LGRs (Hauser et al., 2008; Van Hiel et al., 2012).

LGR1 from *D. melanogaster* is activated by a heterodimer formed by GPA2 and GPB5 (Sudo et al., 2005) which are produced in neuroendocrine cells of the ventral nervous system (Sellami et al., 2011). As is the case for the vertebrate glycoprotein hormones, both of these subunits are cystine knot proteins with complex three dimensional structures (Vitt et al., 2001). Based on transcript studies, *dLgr1* gene expression has been detected throughout all developmental stages of the fruit fly (Hauser et al., 1997; Graveley et al., 2011). In wandering larvae and adults, high transcript abundance has been reported for the hindgut and the salivary glands (Chintapalli et al., 2007).

#### Type B LGRs (CG8930 Orthologs)

LGRs from type B feature 16–18 LRRs, about twice the number found in the other two types (Van Hiel et al., 2012). In vertebrates, three type B LGRs can be identified, whereas in insect genomes only one type B has been found. The *D. melanogaster* member of the type B LGRs, LGR2 (*rk*) was cloned in 2000 (Eriksen et al., 2000) and was activated by bursicon (Luo et al., 2005; Mendive et al., 2005). Analogous to the known ligands of the LGR type A receptors, this hormone is a heterodimer of cystine knot glycoproteins. The bursicon hormone itself had already been described in the 1960s (Fraenkel et al., 1966), but it took until 2004 before its sequence was unraveled (Dewey et al., 2004; Honegger et al., 2004). Bursicon was found to induce the hardening and darkening of the cuticle of newly eclosed adult flies as well as the expansion of the wings (Luo et al., 2005; Mendive et al., 2005). More recently, bursicon has been shown to be responsible for the maturation of the wing, driving the epithelial-mesenchymal transition of the wing epithelial cells (Natzle et al., 2008), but the authors reported that apoptosis associated with wing maturation was not bursicon-regulated in contrast to previous results (Kimura et al., 2004). With regard to wing expansion, it has been proposed that the bursicon secreting neurons in the abdominal ganglion are responsible for neurohemal release, whereas the bursicon-positive neurons in the subesophageal ganglion would orchestrate wing expansion behavior (Peabody et al., 2008). Also, there are indications that bursicon is released preceding the initiation of larval ecdysis and that it is responsible for tanning the pupal case (Loveall and Deitcher, 2010). Additionally, recent data indicate that homodimers of the bursicon α- and β-subunits induced innate immunity genes in the fruit fly (An et al., 2012).

In addition to *D. melanogaster*, LGR2 homologues have been identified in representatives of most insect orders including in *A. mellifera*, *T. castaneum*, and *A. pisum* (Hauser et al., 2006, 2008, 2010; Van Hiel et al., 2012). Interestingly, in *A. mellifera* a single gene was found to encode bursicon. This protein features two cystine knot domains similar to the dimer of two cystine-knot proteins as is the case in the fruit fly and the silk moth (Mendive et al., 2005).

#### Type C LGRs (CG31096/CG6857 and CG34411/CG4187 Orthologs)

In contrast to the vertebrate type C LGRs which are activated by members of the insulin-relaxin peptide family, in insects these receptors are largely uncharacterized. In *D. melanogaster*, two members of the type C LGRs can be identified, dLGR3 and dLGR4. In contrast, in *A. mellifera* and *T. castaneum*, only one receptor has been found which is, respectively, most closely related to dLGR3 and dLGR4 (Hauser et al., 2008). The ligands of these receptors are still unknown.

### Myosuppressin receptors (CG8985 and CG43745/CG13803 Orthologs)

Myosuppressins have a conserved C-terminal FLRFamide. The first myosuppressin was isolated from *L. maderae* (Holman et al., 1986). Myosuppressins inhibit gut contractions and regulate heart contractions (Holman et al., 1986; Lange and Orchard, 1998; Wasielewski and Skonieczna, 2008; Maestro et al., 2011). They also contribute to the regulation of digestive processes by controlling the release of several digestive enzymes in the alimentary canal (Harshini et al., 2002; Hill and Orchard, 2005). Furthermore, myosuppressins inhibit food uptake and thus seem to classify as anorexic factors (Matthews et al., 2008; Vilaplana et al., 2008; Down et al., 2011; Nagata et al., 2011). The first putative myosuppressin receptor was characterized in *L. migratoria*. Cold competition binding studies and kinetic binding assays with a radiolabeled ligand were used to calculate the dissociation constant of the receptor (Kwok and Orchard, 2002). *D. melanogaster* possesses two myosuppressin receptors, DMSR-1 (CG8985) and DMSR-2 (CG43745/CG13803), and were activated by *D. melanogaster* myosuppressin in a dose-dependent manner. Another myosuppressin receptor was characterized in *A. gambiae* (Schöller et al., 2005). Additional myosuppressin receptors have been annotated in *A. aegypti*, *A. mellifera*, *N. vitripennis*, *T. castaneum*, *A. pisum*, and *B. mori*. DMSR-2 is expressed in the head and the body and possibly regulates the actions of myosuppressin on visceral muscles. DMSR-1 is only expressed in the head (Egerod et al., 2003a). Myosuppressin receptors are not evolutionary related to FMRFamide receptors and both represent two separately evolved signaling systems, despite the resemblance of their ligands (Schöller et al., 2005).

### Neuropeptide F receptors (CG1147 Orthologs)

Invertebrate neuropeptide F (NPF) peptides are structural homologues of the vertebrate NPY family. The *Drosophila* NPF neuropeptide was the first full length member of the NPY/NPF family identified in insects (Brown et al., 1999). The insect NPF neuropeptides are characterized by the consensus sequence x_n_PxRx_n_YLx_2_Lx_2_YYx_4_RPRFamide (Nässel and Wegener, 2011). NPF is involved in various processes in *Drosophila* like foraging, feeding, alcohol sensitivity, stress, aggression, reproduction, learning, and locomotion (Shen and Cai, 2001; Wu et al., 2003, 2005a,b; Wen et al., 2005; Lee et al., 2006; Dierick and Greenspan, 2007; Lingo et al., 2007; Chen et al., 2008; Krashes et al., 2009; Xu et al., 2010; Hermann et al., 2012; Shohat-Ophir et al., 2012, for a review, see: Nässel and Wegener, 2011). In several other insects NPF is also (predicted to be) involved in feeding behavior (Zhu et al., 1998; Stanek et al., 2002; Garczynski et al., 2005; Gonzalez and Orchard, 2008, 2009; Nuss et al., 2008, 2010; Ament et al., 2011; Huang et al., 2011b). NPF has also an effect on cardiac activity in the blowfly *Protophormia terraenovae* (Setzu et al., 2012). The *Drosophila* NPF receptor was characterized by means of a radioreceptor approach. The signaling pathway probably acts via Gi and adenylate cyclase as determined by NPF-induced inhibition of forskolin-stimulated cAMP production (Garczynski et al., 2002). The NPF receptor was also characterized in *Anopheles* (Garczynski et al., 2005) and has been predicted in several other insects like *Bombyx* and *Tribolium* (Hauser et al., 2008; Yamanaka et al., 2008; Fan et al., 2010). The proposed *Nasonia* NPF receptor (Hauser et al., 2010) is more likely to be a short NPF receptor; consequently there is probably no NPF receptor present in *Nasonia*. Expression of the *Drome*-NPF receptor was observed in cells of the midgut and numerous neurons in the brain and ventral nerve cord of the third instar larva (Garczynski et al., 2002). The NPF receptor was also located in the adult brain (Wen et al., 2005; Krashes et al., 2009). The *Anoga*-NPF receptor was detected in all life stages except for eggs (Garczynski et al., 2005).

### Pigment dispersing factor receptors (CG13758 Orthologs)

The first pigment dispersing factor (PDF) neuropeptide in insects was characterized in *Romalea microptera* (Rao et al., 1987). The best know function of PDF is its role in the circadian clock as a network coordinator, output factor and regulator of its plasticity similar to the vertebrate vasoactive intestinal peptide (VIP). Further processes that where associated with PDF are activity, reproduction, arousal, and geotaxis (for a review, see: Meelkop et al., 2011). Recently, also a role for PDF in the control of visceral physiology in *Drosophila* was described, thereby extending the similarities between fly PDF and VIP in mammals (Talsma et al., 2012). In 2005, three research groups simultaneously identified the PDF receptor in *Drosophila*. Mertens et al. (2005) found the receptor to be specifically responsive to PDF and to couple with Gs, leading to an elevated cAMP concentration upon receptor activation. Mutants showed an aberrant behavioral rhythmicity and a severe negative geotaxis. In a large-scale temperature preference behavior screen in *Drosophila*, Hyun et al. (2005) identified a mutant that preferred colder temperatures during the night and named it *han* (Korean for cold). *Han* seems to be a mutant of a P element controlling the CG13758 gene. But mutations in the latter gene did not cause temperature preference difference. Instead it shows arrhythmic circadian behavior in constant darkness as seen in *pdf* null mutants. PDF specifically binds to S2 cells expressing HAN and thereby elevates the cAMP level. The third research group also identified a mutant with the same disrupted circadian behavior as *pfd* mutants and named it *groom-of-PDF* (*gop*) (Lear et al., 2005). Later studies showed, however, that only the advanced evening activity is common with the *pdf* mutants. *pdfr* mutants, in contrast to *pdf* mutants, did have a morning peak (Im and Taghert, 2010). There are several indications that *pdfr* is regulated at steady-state level by the clock gene *period* (Lear et al., 2005; Mertens et al., 2005). Localization studies showed PDFR expression in the brain and visual system in close correspondence to PDF expression. PDFR expression shows also similarities to the clock pacemaker network of neurons. Furthermore expression is found dispersed in the anterior and posterior surfaces of the central brain and subesophageal ganglion (Shafer et al., 2008; Im and Taghert, 2010). In embryos no expression was noticed (Hyun et al., 2005). *Drosophila* is the only insect where the PDFR has been deorphanized so far. However, homologous sequences are found in many insects like several *Drosophila* species, *A. gambiae*, *A. mellifera*, *N. vitripennis*, *B. mori*, and *T. castaneum*.

### Proctolin receptors (CG6986 Orthologs)

Proctolin or RYLPT is a myo- and neurostimulatory neuropeptide of which the appearance seems to be restricted to arthropods (Starratt and Brown, 1975; Nässel, 2002). It stimulates or potentiates muscle contraction, is cardio-acceleratory and acts as a neurohormone (Orchard et al., 1989; Lange, 2002; Clark et al., 2006; Lange and Orchard, 2006; Nässel and Winther, 2010). The *Drosophila* gene for the proctolin receptor was identified and cloned (Egerod et al., 2003b; Johnson et al., 2003a,b; Taylor et al., 2004; Orchard et al., 2011). When the receptor was stably expressed in CHO/Gα_16_ cells, a dose-dependent response was measured for proctolin (Egerod et al., 2003b). In competition-based studies, the proctolin receptor binds proctolin with high affinity (Johnson et al., 2003a). The proctolin and/or proctolin receptor gene was found in the genomes of only a few insect species, including *T. castaneum*, *T. molitor*, *P. humanus*, and *A. pisum* (Hauser et al., 2008; Li et al., 2008; Weaver and Audsley, 2008; Huybrechts et al., 2010). No proctolin gene has been identified in genomes of *A. aegypti*, *A. gambiae*, *A. mellifera*, *N. vitripennis, B. mori*, or *Acromyrmex echinatior* and three other ant species (Hauser et al., 2006, 2010; Roller et al., 2008; Predel et al., 2010; Nygaard et al., 2011), where proctolin and its receptor are now considered absent.

### Pyrokinin receptors (CG8784, CG8795 and CG9918 Orthologs)

Pyrokinins are characterized by the C-terminal sequence FXPRLamide (X = S, T, K, A, or G) (Holman et al., 1986; Predel et al., 2001). They are involved in the stimulation of gut motility, the production and release of sex pheromones, diapause, and pupariation (Holman et al., 1986; Predel et al., 2001; Nässel, 2002; Altstein, 2004; Verleyen et al., 2004a; Homma et al., 2006). The pyrokinins can be subdivided into two groups, pyrokinin-1 (C-terminus WFGPRLamide) and pyrokinin-2 (C-terminus PFKPRLamide) (Cazzamali et al., 2005). The first identified insect pyrokinin receptors were those of *D. melanogaster*, where three pyrokinin receptors occur. CG9918 seems to be specific for pyrokinin-1 and CG8784 and CG8795 for pyrokinin-2 (Park et al., 2002; Rosenkilde et al., 2003; Cazzamali et al., 2005). Two pyrokinin receptors were cloned and pharmacologically characterized in *A. gambiae*, one being more specific for pyrokinin-1, the other for pyrokinin-2 (Olsen et al., 2007). The pyrokinin-2 receptor orthologue of *Helicoverpa zea* expressed in *Spodoptera frugiperda* (Sf) 9 cells also responded to pheromone biosynthesis-activating neuropeptide (PBAN) in the low nanomolar range (Choi et al., 2003).

*A. mellifera* has two pyrokinin receptor orthologs, but since they both have the same sequence identities (55–56%) to the *Drosophila* genes, it is difficult to classify them as pyrokinin-1 or -2 receptors (Hauser et al., 2006). The *T. castaneum* genome contains probably three pyrokinin receptors, which are currently classified according to their highest amino acid residue identities (Hauser et al., 2008). Pyrokinin receptors have been found in all insects so far, but it is difficult to classify them as pyrokinin-1 or -2 receptors (Jurenka and Nusawardani, 2011). This will remain problematic until *in vivo* studies using genetics will have solved this issue (Melcher et al., 2006).

### RYamide receptors (CG5811 Orthologs)

In 2010, a new class of neuropeptides was discovered from the genome of *N. vitripennis*. These RYamides are characterized by the C-terminal motif FFxxxRYamide (Hauser et al., 2010). Thereupon, RYamides were identified for all insects with a sequenced genome, except for some ant species (Hauser et al., 2010; Nygaard et al., 2011). Recently, the RYamide receptors for *D. melanogaster* and *T. castaneum* were characterized using CHO/Gα_16_ cells. Both *Drosophila* RYamides were capable of activating the receptor in the nanomolar range. For *T. castaneum* it was observed that *Trica*-RYamide-2 is somewhat more potent than *Trica*-RYamide-1 to activate the receptor (Collin et al., 2011; Ida et al., 2011a). Although the *Drosophila* receptor was also activated by high concentrations of mammalian NPY and NPYY (Li et al., 1992), a phylogenetic analysis seems to indicate that there is no significant structural relationship between NPY and RYamide receptors (Collin et al., 2011). A first study to unravel the function of the RYamides was performed in *Phormia regina*. Injections of *Drosophila* RYamide-1 attenuate the feeding motivation of these flies (Ida et al., 2011a). The receptor is mainly expressed in the hindgut, while it is not, or hardly present in other investigated tissues in *Drosophila* males and females. This strengthens the hypothesis that the signaling system has a role in digestion, or maybe water reabsorption (Collin et al., 2011; Ida et al., 2011a).

### Sex peptide/myoinhibiting peptide receptor (CG16752/CG12731 Orthologs)

SP induces the post-mating effects that occur in female fruit flies (Kubli and Bopp, 2012). It is produced in the male accessory glands and transferred with the seminal fluid during copulation. It induces egg laying and loss of receptivity for additional mating (Chen et al., 1988), alters the female's sleep pattern (Isaac et al., 2010) and provokes antimicrobial peptide expression (Peng et al., 2005; Domanitskaya et al., 2007). Additionally, the food uptake and preference of females is altered after copulation (Carvalho et al., 2006; Barnes et al., 2007; Kubli, 2010; Ribeiro and Dickson, 2010; Vargas et al., 2010). The SP receptor (SPR) from *D. melanogaster* has been characterized and homologues of this receptor were identified in various insects with the exception of Hymenoptera (Yapici et al., 2008; Kim et al., 2010). Expression of this receptor is found in the female reproductive organs, especially the spermatheca, and the central nervous system of both males and females in very similar patterns (Yapici et al., 2008; Häsemeyer et al., 2009; Poels et al., 2010).

In addition to SP, the related ductus ejaculatorius peptide (DUP) 99B (Saudan et al., 2002) can activate SPR (Yapici et al., 2008). Although both SP and DUP99B have only been identified in *Drosophila* species, they can also elicit physiological responses in the lepidopteran *Helicoverpa armigera* (Fan et al., 1999). As SP and DUB99B so far have only been found in most—not all—*Drosophila* species, the receptor's evolutionary conservation was a puzzle that was only recently solved. SPR can be activated not only by SP, but also by myoinhibiting peptides (MIPs, also known as B-type ASTs) (Kim et al., 2010; Poels et al., 2010). These neuropeptides show the same evolutionary conservation as SPR and therefore likely correspond to the ancestral ligands of SPR. MIPs display a characteristic WX_6_Wamide C-terminal motif and were first purified from *L. migratoria* (Schoofs et al., 1991), but members of the neuropeptide family were also identified in other species such as *Gryllus bimaculatus*, *D. melanogaster*, and *R. prolixus* (Lorenz et al., 1995; Williamson et al., 2001; Lange et al., 2012). MIPs display myoinhibiting activity in visceral muscle preparations *in vitro* (Schoofs et al., 1991; Blackburn et al., 1995, 2001; Predel et al., 2001). In *G. bimaculatus*, they inhibit JH biosynthesis (Lorenz et al., 1995), and in *D. melanogaster* and *M. sexta*, MIP may silence neurons that are not required during the ecdysis program (Kim et al., 2006a,b). Evidence from *B. mori* indicates that expression of the MIP receptor is strongly upregulated following a sudden decline of the 20-hydroxyecdysone titer. Therefore, MIP receptor signaling may be involved in the fine-tuning of ecdysteroid titers (Yamanaka et al., 2010).

### Short neuropeptide F receptors (CG7395/CG18639 Orthologs)

sNPF neuropeptides were first identified in *A. aegypti* and indicated as “*Aedes* head peptides” (Matsumoto et al., 1989). Nowadays, sNPFs are predicted in all insect with a sequenced genome and they are characterized by the C-terminal consensus sequence xPxLRLRFamide (Nässel and Wegener, 2011). The main functions of sNPF seem to be linked to the regulation of feeding behavior (Lee et al., 2004, 2008, 2009; Chen and Pietrantonio, 2006; Kahsai et al., 2010; Ament et al., 2011; Lu and Pietrantonio, 2011; Nagata et al., 2011, 2012b; Root et al., 2011; Hong et al., 2012; Mikani et al., 2012). Other processes in which sNPF is probably involved in are diapause, learning behavior, ovarian growth stimulation, metabolic stress, cardiac activity, the circadian rhythm, and the regulation of hormone production and hormonal release (Schoofs et al., 2001; Huybrechts et al., 2004; Johard et al., 2008; Nässel et al., 2008; Kahsai et al., 2010; Lu and Pietrantonio, 2011; Kapan et al., 2012; Setzu et al., 2012; for a review, see: Nässel and Wegener, 2011). As previously discussed in section “Allatotropin Receptors (NP_001127714 Orthologs),” sNPF peptides may also possess allatotropic activity (Yamanaka et al., 2008). The first sNPF receptor was identified in *Drosophila* and all four predicted *Drosophila* sNPF peptides activate the receptor in physiological concentrations (Mertens et al., 2002; Feng et al., 2003). Also in *Solenopsis invicta* (Chen and Pietrantonio, 2006), *A. gambiae* (Garczynski et al., 2007), and *B. mori* (Yamanaka et al., 2008) the sNPF receptor has been deorphaned. When co-expressed in *Xenopus* oocytes, the *Drosophila* sNPF receptor activates exogenously expressed inwardly rectifying K^+^ channels (Reale et al., 2004). The sNPF receptor is present in a limited number of neurons in the nervous system of all developmental stages. Throughout development, the receptor is also expressed in peripheral tissues including the gut, Malpighian tubules, fat body, and ovaries as has been shown in various insects (Mertens et al., 2002; Feng et al., 2003; Chen and Pietrantonio, 2006; Garczynski et al., 2007; Yamanaka et al., 2008; Lu and Pietrantonio, 2011; Kahsai et al., 2012; Nagata et al., 2012b).

### Sifamide receptors (CG10823 Orthologs)

SIFamides are highly conserved during evolution and have been isolated from various insects (Verleyen et al., 2004b; Audsley and Weaver, 2006). SIFamide is present in four neurons in the insect pars intercerebralis and this specific pattern suggests a neuromodulatory role in combining visual, tactile and olfactory input. Targeted cell ablation and RNAi has revealed that SIFamide modulates sexual behavior in fruit flies (Terhzaz et al., 2007). The *Drosophila* SIFamide receptor is activated by the SIFamide (Jørgensen et al., 2006). The identification of well-conserved SIFamide receptor orthologs in all insects with a sequenced genome, suggests that SIFamide signaling regulates an essential function in arthropods (Hauser et al., 2006, 2008; Jørgensen et al., 2006; Verleyen et al., 2009).

### Sulfakinin and cholecystokinin (CCK)-like receptors (CG32540/CG6894/CG6881 and CG42301/CG6857 Orthologs)

Sulfakinins (SKs) are the insect homologues of the vertebrate cholecystokinin (CCK) and gastrin neuropeptides (Nachman et al., 1986a,b; Staljanssens et al., 2011). They are named after the sulfated tyrosyl residue in their active core sequence YGHMRFamide that is usually required for biological activity. The first insect SKs were isolated from *L. maderae* and stimulated hindgut contractions (Nachman et al., 1986a,b). Peptidomic techniques elucidated SK peptides in all major insect orders (Verleyen et al., 2004a; Li et al., 2008; Ons et al., 2009; Hauser et al., 2010; Huybrechts et al., 2010; Audsley et al., 2011; Zoephel et al., 2012). SK regulates food uptake and works as a satiety factor that inhibits feeding in several insect species (Wei et al., 2000; Maestro et al., 2001; Downer et al., 2007; Meyering-Vos and Müller, 2007). Drosulfakinins are coreleased with ILPs and influence food choice in *D. Melanogaster* (Söderberg et al., 2012). It stimulates hindgut contractions (Nachman et al., 1986a,b; Marciniak et al., 2011), but inhibits contractions of the heart, oviduct and ejaculatory duct (Marciniak et al., 2011). Only one insect SK receptor, the *D. melanogaster* SK receptor 1 (*Drome*-SKR1) has been deorphanized so far. It is activated by [Leu^7^]-*Drome*-SK-1 at nanomolar concentrations. [Leu^7^]-*Drome*-SK-1 was tested instead of the endogenous [Met^7^]-*Drome*-SK-1 for stability reasons. The sulphate residue is essential for high-affinity receptor binding in all tested cellular assays (Kubiak et al., 2002). SK receptors are widespread in insects: *T. castaneum* and *A. gambiae* have two SK receptors, while *A. aegypti*, *A. mellifera*, and *B. mori* contain at least one.

*Drosophila* contains a second, recently characterized, SK receptor, the CCK-like receptor (Chen et al., 2012). As both SK receptors probably arose through a gene duplication and because of the high homology between the two, it is likely that they also display similar ligand specificity (Hewes and Taghert, 2001; Kubiak et al., 2002). Both, CCKLR and DSK are strong positive growth regulators of the *D. melanogaster* larval neuromuscular junction (Chen and Ganetzky, 2012), by signaling via the cAMP-protein kinase A (PKA)-CRE binding protein (CREB) pathway, known for its role in structural synaptic plasticity in learning and memory (Chen and Ganetzky, 2012). A β-arrestin translocation assay in HEK cells was used to show that sulfated drosulfakinins are the endogenous ligands for CCKLR-17D1. Binding of DSK-1S or DSK-2S to the receptor promotes larval locomotion and evokes stress-induced larval escape behavior (Chen et al., 2012).

### Tachykinin receptors (CG6515 and CG7887 Orthologs)

Insect tachykinins differ from mammalian tachykinins by their C-terminal consensus sequence, which is FX_1_GX_2_Ramide, rather than FXGLMamide as in mammals. There are many different tachykinin isoforms in each insect, which are all encoded by a single gene (Siviter et al., 2000). They play various roles in neuronal signaling and gut activity (Vanden Broeck, 2001; Nässel, 2002; Coast and Garside, 2005; Predel et al., 2005; Van Loy et al., 2010). The first insect GPCR capable of sensing tachykinin-related neuropeptides was cloned from *Drosophila* and is termed *Drosophila* tachykinin receptor (DTKR and CG7887) (Li et al., 1991). *Drosophila* tachykinin-related neuropeptides (*Drome*-TKs) are the endogenous ligands of DTKR and dose-dependently increased intracellular Ca^2+^ concentrations, as well as cyclic AMP levels, when applied on DTKR-expressing HEK293 or S2 cells (Birse et al., 2006; Poels et al., 2007). DTKR is involved in the regulation of insulin signaling and the olfactory sensory processing in the antennal lobe (Ignell et al., 2009; Birse et al., 2011).

A second tachykinin receptor in *Drosophila* is the neurokinin receptor (NKD and CG6515) (Monnier et al., 1992). *Drome*-TK-6 (with an Ala instead of Gly) is the only known fly neuropeptide with clear agonist activity on NKD-expressing cells (Poels et al., 2009), which suggests that NKD is able to discriminate between Ala- and Gly-containing isoforms of tachykinin ligands, a feature that does not apply to DTKR (Van Loy et al., 2010). A similar tachykinin receptor has been cloned from *Stomoxys calcitrans* (STKR) (Guerrero, 1997). Its endogenous ligand, *Stoca*-TK, which contains an Ala-residue instead of the highly conserved Gly-residue, behaves as a partial agonist (Poels et al., 2009; Van Loy et al., 2010).

A putative tachykinin receptor has been cloned from brain tissue of *L. maderae* (Johard et al., 2001). One or two tachykinin receptor orthologs have been identified in all insects with a sequenced genome (Hauser et al., 2006, 2008), pointing at an indispensable role of these proteins.

### Trissin receptor (CG34381/CG14003 Orthologs)

Trissin is a recently identified neuropeptide that contains six Cys residues which form three intramolecular disulfide bridges. Trissin has been shown to activate the *D. melanogaster* GPCR CG34381 stably expressed in CHO/Gα_16_ cells at picomolar concentrations. Given the toxic and antimicrobial properties of many Cys containing neuropeptides, one hypothesis is that trissin may have an antimicrobial function. Transcript profiling data for trissin and its receptor on the other hand indicated that transcripts for both were present in the central nervous system of third instar larvae and adults, suggesting that trissin might be a neuropeptide (Ida et al., 2011b). Neuropeptides with high sequence similarity to trissin have been found in several *Drosophila* species and in three mosquitoes, *A. aegypti*, *A. gambiae*, and *C. quinquefasciatus* as well as in *B. mori* (Ida et al., 2011b). In *A. mellifera*, raalin displayed sequence similarity with trissin but this neuropeptide only features 5 Cys residues (Kaplan et al., 2007). Since currently no signal sequence or C-terminal processing sites have been identified for this neuropeptide, its sequence may still be incomplete.

## Remaining orphan *Drosophila* receptors

At least 14 GPCRs predicted to have a neuropeptide as cognate ligand are still orphan and include CG4313, CG12290, CG32547/CG12610, CG13229, CG13995, CG33696/CG16726, CG33639/CG5936, CG30340, and CG13575.

The orphan receptor *hector* (CG4395) is involved in the regulation of *Drosophila* male courtship behavior. It is expressed in numerous brain cells, mainly in the mushroom bodies, the central complex and, at lower levels, in a subset of glomeruli in the antennal lobes. However, only those cells that co-express *fruitless* (*fru*—one of the two main regulators of male courtship behavior besides *doublesex*) and *hector* are critical for male courtship (Li et al., 2011b).

The *moody* gene (CG4322) encodes two splice variants, Moody-α and Moody-β that differ in their long carboxy-terminal domains. Both receptors are coexpressed in glial cells that surround and insulate the nervous system, which is required for the formation and maintenance of the *Drosophila* blood-brain barrier (Bainton et al., 2005; Schwabe et al., 2005; Hatan et al., 2011). The moody receptors are also involved in drug sensitivity (Bainton et al., 2005; for a review, see: Daneman and Barres, 2005).

The receptor encoded by *trapped in endoderm 1* (*tre1*, CG3171) is a functional analog of the CXCR4 receptor of vertebrates, which is involved in tumor metastasis (Kamps et al., 2010). Tre1 is essential for the transepithelial migration of *Drosophila* germ cells from the posterior midgut toward the gonads (Kunwar et al., 2003). The receptor probably plays a role in three phases of early migration: polarization of germ cells, dispersal into individual cells, and transepithelial migration (Kunwar et al., 2008). Tre1 also regulates the relative orientation of cortical polarity in embryonic *Drosophila* neural stem cells (neuroblasts) (Yoshiura et al., 2012).

## Methuselah (CG6936) and methuselah-like receptors

The *methuselah* (*mth*) gene encodes a family B GPCR and is involved in stress response and biological ageing in *Drosophila* (Lin et al., 1998). Also 15 *methuselah-like* (*mthl*) relatives were identified in *Drosophila*, most of them characterized by a unique motif in the extracellular domain consisting of up to ten cysteine residues and several glycosylation sites. (West et al., 2001; Patel et al., 2012). Two peptides were identified capable of activating the Mth receptor in a dose-dependent manner. These peptides correspond with the splice variants A and B of the *Drosophila* gene *stunted* (*sun*), which codes for the ε-subunit of mitochondrial ATP synthase (Cvejic et al., 2004; Kidd et al., 2005). The EC_50_ values for both polypeptides, however, were quite high with 4 μM for Sun A and 3.8 μM for Sun B. A second splice variant of the receptor was activated in somewhat lower doses with an EC_50_ value of 0.6 μM for Sun A and Sun B. The *stunted* gene was also shown to be involved in ageing and oxidative stress (Cvejic et al., 2004). Later on, the *Drosophila* SP and a non-physiological peptide with a randomly generated sequence, the serendipitous peptide activator of Mth (SPAM), were also identified as agonists of the Mth receptor. The peptides share almost no sequence homology with Sun A and B, indicating the promiscuity of Mth for activation (Ja et al., 2009). However, *mth* mutants do not affect behaviors controlled by SP, so it is doubtful that activation of the Mth receptor by these ligands is of any biological significance (Ja et al., 2009). An extensive developmental expression and sequence divergence study was performed by Patel et al. (2012). More studies are needed to affirm which are the cognate ligands of the Mth receptor and to unravel the physiological roles of the methuselah-like receptors.

## Remaining orphan neuropeptides

For several neuropeptides, including the ion transport peptides (ITPs), neuroparsins, orcokinins, and amnesiac, the cognate receptor is unknown at the moment. ITPs function as antidiuretic hormones in locusts (Audsley et al., 1992; Phillips et al., 2001). ITPs are found in the genomes of many insect orders including dipterans, lepidopterans, and coleopterans (Dircksen et al., 2008; Begum et al., 2009; Dircksen, 2009).

Neuroparsins are pleiotropic neuropeptides and are inter alia involved in reproduction and serve as molecular markers of the process of phase transition in locusts (Brown et al., 1998; Girardie et al., 1998; Claeys et al., 2005b, 2006; Badisco et al., 2007). It is noteworthy that in the genus *Drosophila*, the gene coding for the neuroparsins is absent from the *melanogaster* subgroup of the subgenus *Sophophora*, although present in other species of the genus (Veenstra, 2010).

Insect orcokinins were first identified in *B. germanica* and *S. gregaria* (Pascual et al., 2004; Hofer et al., 2005) and were subsequently detected in various other insects, excluding *Drosophila* and *Tribolium* (Roller et al., 2008). A study in *L. maderae* indicates that orcokinins are involved in circadian behavior (Hofer and Homberg, 2006). In *B. mori* it was demonstrated that orcokinins act as prothoracicotropic factors and as such are involved in ecdysteroidogenesis (Yamanaka et al., 2011). Recently, a new family of neuropeptides was discovered in *R. prolixus*, named Orcokinin B, because it arises due to alternative splicing of the *orcokinin* gene. Orcokinin B expression is observed in several insects, except *Drosophila* spp. and *A. pisum* (Sterkel et al., 2012).

The *amnesiac* (*amn*) gene, which encodes a putative neuropeptide precursor (Feany and Quinn, 1995; Moore et al., 1998), is important for stabilizing olfactory memory, and is involved in various aspects of other associative and non-associative learning (Quinn et al., 1979; Gong et al., 1998; DeZazzo et al., 1999; Keene et al., 2004, 2006; Yu et al., 2006; Motosaka et al., 2007). Additional studies have indicated that *amn* is also involved in ethanol sensitivity, sleep, temperature preference behavior and nociception (Moore et al., 1998; Hong et al., 2008; Liu et al., 2008; Aldrich et al., 2010). The *amn* gene was found to code for neuropeptides closely related to the vertebrate pituitary adenylate cyclase-activating polypeptide (PACAP) and submammalian glucagon/growth hormone-releasing hormone (GHRH) and were shown to possess phylogenetically conserved functions (Hashimoto et al., 2002).

## Discussion and future prospects

This review clearly shows that during the last two decades a tremendous progress has been made on the field of insect neuropeptide signaling systems. This progress is mostly attributable to the increased availability of insect genomes and the advancing fields of genomics and peptidomics. It became clear that several of these systems are well conserved in all insect species, suggesting that they are indispensable in general insect physiological functions. Other neuropeptides and their receptors were apparantly lost during evolution in several insect species or orders, suggesting that they were otiose, or that their functions were taken over by other ligands. To gain more insight into the evolution of neuropeptide GPCRs across the Insecta, more insect genomes need to be sequenced, which may soon be accomplished due to the i5K project (Robinson et al., 2011). But despite the great progression made in insect endocrinology, the knowledge about the functions of many of the neuropeptides and their receptors involved is still scarce. Furthermore, even in *Drosophila*, the preeminent insect model organism, various receptors are still orphan and the physiological roles they play are still a mystery. It may be obvious that a lot of work has to be performed before the functions of the different signaling systems will be clearly understood and to unravel how these systems are intertwined with each other. This information is also necessary to get a better view on the evolutionary origin of the peptide-receptor couples and how they changed during evolution among species. It should be emphasized that sequence similarity between different insects does not necessarily implies functional similarity or *vice versa*. So, it remains a prerequisite to functionally characterize neuropeptide GPCRs in several insect model species. Reverse genetic tools including RNAi, or the application of the fairly new technique of genome editing using engineered zinc finger nucleases (Urnov et al., 2010) are only some of the methods being developed in several insects, which will likely boost GPCR functional research. Nevertheless, cross genome clustering of receptors based on sequence homology may be a good starting point to acquire a better view on their putative functions (Metpally and Sowdhamini, 2005).

The usefulness of research on insect neuropeptide signaling systems goes beyond the world of insects as several mammalian neuropeptides and/or their receptors have orthologs in insects. Well studied examples of such conserved signaling systems are the GnRH (Lindemans et al., 2011; Roch et al., 2011; De Loof et al., 2012), the tachykinin (Pennefather et al., 2004; Van Loy et al., 2010), the NPF/NPY (Nässel and Wegener, 2011), the capa-pyrokinin/neuromedin (Melcher et al., 2006; Terhzaz et al., 2012), the AST C/somatostatin (Birgül et al., 1999; Veenstra, 2009a), the myoinhibiting peptide/galanin (Blackburn et al., 1995), the PDF/VIP (Vosko et al., 2007; Talsma et al., 2012), the diuretic hormone/corticotropin releasing hormone (Lovejoy et al., 2009; De Loof et al., 2012), the diuretic hormone/calcitonin (Zandawala, 2012), and the SK/CCK (Staljanssens et al., 2011) orthologs, meaning that these signaling systems arose before the divergence of the Proto- and Deuterostomia (more than 700 million years ago). This strengthens the reasons to study insect endocrinology as these studies can help and learn vertebrate endocrinologists more about the current vertebrate receptors (Grimmelikhuijzen and Hauser, 2012a).

It should be clear that the importance of neuropeptides and their receptors in insect physiology can hardly be overestimated. The great variety among the neuropeptides and their receptors between different insect species makes them also potential targets for the development of a new generation of insecticides with high species specificity (Grimmelikhuijzen et al., 2007; Bendena, 2010; Grimmelikhuijzen and Hauser, 2012b). Such insecticides would ideally only be harmful for pest insects like insects acting as vectors for diseases or herbivorous insects detrimental for agriculture, while beneficial insects would be unharmed. The development of such new controlling agents is necessary because of the detrimental effects of the currently used insecticides on the environment and their toxicity to non-target organisms. The increasing resistance of pest insects against the used insecticides is also an expanding problem (Casida and Quistad, 1998; Van Hiel et al., 2010). As such, more and more research is performed on neuropeptides in order to develop synthetic ligands that can disturb the proper functioning of neuropeptide signaling systems, provoking detrimental effects on the insect's fitness. Once again we want to emphasize the importance of elucidating the biochemical pathways and the functions of the neuropeptides in order to be able to design these so called peptidomimetics. Hitherto, no neuropeptide-based insecticides are in use, but a lot of progress is made on a number of interesting neuropeptides (Teal et al., 1999; Gäde and Goldsworthy, 2003; Altstein, 2004; Scherkenbeck and Zdobinsky, 2009; Altstein and Nässel, 2010; Nachman and Pietrantonio, 2010).

### Conflict of interest statement

The authors declare that the research was conducted in the absence of any commercial or financial relationships that could be construed as a potential conflict of interest.
